# Research progress on exosomes/microRNAs in the treatment of diabetic retinopathy

**DOI:** 10.3389/fendo.2022.935244

**Published:** 2022-08-09

**Authors:** Si-ru Niu, Jian-min Hu, Shu Lin, Yu Hong

**Affiliations:** ^1^ Department of Ophthalmology, The Second Affiliated Hospital of Fujian Medical University, Quanzhou, China; ^2^ The School of Medical Technology and Engineering, Fujian Medical University, Fuzhou, China; ^3^ Centre of Neurological and Metabolic Research, The Second Affiliated Hospital of Fujian Medical University, Quanzhou, China; ^4^ Group of Neuroendocrinology, Garvan Institute of Medical Research, Sydney, NSW, Australia

**Keywords:** Diabetic retinopathy, pericyte, microRNA, exosomes, treatment

## Abstract

Diabetic retinopathy (DR) is the leakage and obstruction of retinal microvessels caused by chronic progressive diabetes that leads to a series of fundus lesions. If not treated or controlled, it will affect vision and even cause blindness. DR is caused by a variety of factors, and its pathogenesis is complex. Pericyte-related diseases are considered to be an important factor for DR in many pathogeneses, which can lead to DR development through direct or indirect mechanisms, but the specific mechanism remains unclear. Exosomes are small vesicles of 40–100 nm. Most cells can produce exosomes. They mediate intercellular communication by transporting microRNAs (miRNAs), proteins, mRNAs, DNA, or lipids to target cells. In humans, intermittent hypoxia has been reported to alter circulating excretory carriers, increase endothelial cell permeability, and promote dysfunction *in vivo*. Therefore, we believe that the changes in circulating exocrine secretion caused by hypoxia in DR may be involved in its progress. This article examines the possible roles of miRNAs, proteins, and DNA in DR occurrence and development and discusses their possible mechanisms and therapy. This may help to provide basic proof for the use of exocrine hormones to cure DR.

## 1 Introduction

Microvascular complications are one of the most common complications of diabetes mellitus. Persistent high blood sugar levels can harm a range of organs, including the heart, kidneys, and retina. The most common complications of diabetes are microvascular complications, particularly diabetic nephropathy (DN) and retinopathy (1). Diabetic retinopathy (DR) is chronic retinopathy that causes leakage and blockage of retinal blood vessels, which can result in a range of fundus lesions, including microangiomas, hard exudates, cotton spots, neovascularization, vitreous hyperplasia, macular edema, and retinal detachment, among others. In recent years, with the advancement of medical technology, particularly the emergence of antiangiogenic drugs, there have been more options for DR treatment; however, it remains an important cause of impaired vision and even blindness.

The etiology of DR is associated with several morphological alterations, including pericyte loss, basement membrane thickening, increased vascular permeability, and microaneurysms. Pericytes and endothelial cells (ECs) are the most common retinal vascular cells. Communication between these two cell types is important for microvascular stabilization and remodeling. Pericyte loss is an early pathological feature of DR and frequently occurs in diabetic patients and animals (2). These two cell types share the same basement membrane on the vessel wall. There is communication between them due to the discontinuity of the basement membrane. ECs and pericytes communicate through the gap junctions between PEG sockets with other paracrine signaling factors, including growth factors, secreted cytokines, and extracellular secretions (2–5). Because pericytes are an abundant cell type in microvessels, their dysfunction can result in a variety of vascular-related diseases, including stroke and renal infarction, among others. Studies have shown that diseases associated with the diabetic retina are closely associated with pericytes. For example, pericytes create cPWWP2A (circ RNAs - PWWP2A), which is subsequently transferred to ECs *via* exosomes (5). CPWWP2A silencing can exacerbate diabetic retinal microvascular damage and dysfunction, such as pericyte loss, acellular capillary microaneurysm, vascular leakage, and inflammation (5).

Exosomes refer to small membrane vesicles containing complex RNAs and proteins. Under normal and pathological conditions, many cells secrete exosomes. They are primarily sourced in the multivesicles formed by the invagination of intracellular lysosomal granules, and the outer membranes of the multivesicles are released into the extracellular matrix following fusion with the cell membrane. Exosomes are considered to be membrane vesicles that are secreted exclusively and play a role in intercellular communication. Studies have shown that exosomes can maintain stable blood sugar levels in the body through multiple mechanisms and slow the development of diabetes and the associated microvessel formation, reducing the progression of this diabetic complication. However, the specific mechanism of exosome action remains unclear (1, 2). Many biological effects of exosomes are expressed by microRNA (miRNA), and miRNAs regulate different pathological alterations during DR, which include cell proliferation, apoptosis, inflammation responses, microcirculation impairments, oxidative stress, and cellular death by controlling the key molecules, particularly vascular endothelial growth factor (VEGF) (6). Therefore, miRNA antagonists or mimics as a novel class of drugs could be potentially helpful to control the occurrence and progression of pathological changes during DR.

This article examines the role and possible mechanisms of DR and the occurrence of exosomes and their possible use in its treatment. To reverse the chronic consequences of DR, we might use exosome substances to treat this disease, for example, miRNA-21 and miR-200a-3p.

## 2 Diabetic retinopathy and pericytes

DR is characterized by a severe deterioration of the retinal microvasculature, resulting in hypoperfusion, increased capillary permeability, abnormal proliferation of retinal blood vessels, and ultimately even blindness (7). DR can be classified into two types according to fundus changes: non-proliferative and proliferative phases (7, 8). The non-proliferative phase is confined to the retina; blood vessels undergo microaneurysms and bleeding and display vascular instability, macular edema, basement membrane thickening, and vascular degeneration (9). By comparison, the proliferative phase is characterized by neovascularization. New blood vessels are prone to rupture, which may eventually lead to retinal bleeding and detachment (8).

Although DR is primarily considered to be a disease caused by decreased EC function, there is significant evidence from animal studies that its pathogenesis begins with pericyte loss. Studies of diabetic complications in humans have shown that pericyte exfoliation in DR is associated with microvascular lesion development, including microaneurysms, acellular capillaries, vascular distortion, increased permeability, and capillary perfusion (4). The early pericyte loss is rapidly accompanied by EC loss and capillary network collapse, resulting in reduced retinal blood flow. It is proposed that the initial pericyte loss is driven by angpt-2 (10). The regeneration and plasticity of pericytes allow possible treatment of diseases associated with vascular malnutrition, including muscular dystrophy, ischemic stroke, and DR. To facilitate intercellular communication, the tight binding of pericytes and ECs occurs through direct contact, with ion exchange by gap junctions such as connexin43 (11) and the exchange of other paracrine molecules such as cathepsin D (12) and sphingosine 1-phosphate (13). Pericytes may serve as targets to treat microvascular diseases such as diabetic pathological angiogenesis and complications (14).

## 3 Effects of exosomes on diabetic retinopathy

Exosomes are released into the extracellular space from many cell types. These exosomes are broadly distributed in the body fluids. In recent years, mRNA and miRNA have been identified *in vitro*, which can be absorbed by nearby or distant cells, and regulate the receptor cells, thus playing a role in the occurrence and development of related diseases.

### 3.1 Exosomes

#### 3.1.1 Concept and classification of exosomes

Exosomes currently refers specifically to discoid vesicles with a diameter of 40–100 nm. They are common membrane-bound nanovesicles which transport proteins, lipids, DNA, mRNA, and miRNA among other biomolecules. They are initially formed by endocytosis. Above all, internalization of the cell membrane produces endosomes. Subsequently, many small vesicles are formed in the inner body through the invaginated part of the inner body membrane. Such endosomes are termed multivesicular bodies (MVBs). Finally, MVBs fuse with the cell membrane, releasing endosome vesicles outside the cell as exosomes. Exosomes are produced by cells *via* exocytosis and are taken up by target cells. They transport substances and messages between cells through the circulation of body fluids. Therefore, exosomes play a role in different physiological and pathological processes in the human body (15, 16). Many biological effects of exosomes are expressed through miRNAs. miRNAs are a class of endogenous short non-coding single-stranded RNA molecules of 19–23 nucleotides in length which are from genome regions that do not code for proteins (17). They can be found in human fluids in a stable state, according to increasing data. Extracellular miRNAs can be loaded into high-density lipoprotein or bound by argonaute-2 protein outside vesicles, in addition to being packed into exosomes or microvesicles. All three mechanisms protect miRNAs against degradation and ensure their long-term stability (16). miRNAs negatively regulate the expression levels of target genes and confer characteristic changes on them, playing a regulatory role in almost all cellular processes. When miRNAs are analyzed as exosome miRNAs rather than intracellular miRNAs, researchers discovered a new role in certain cases, being exported inside extracellular vesicles, with Toll-like receptor (TLR)–binding miRNA released by cells from injured or stressed tissues able to reach the endosomal compartment and propagate inflammatory signals in distant recipient cells (17). Exosomal miR-21 and miR-29a were initially revealed to have the ability to attach to TLRs and activate immune cells, in addition to their traditional action of targeting miRNA (18). The quantity and composition of secreted miRNAs vary between diseased and healthy individuals (16, 19–21). To date, hundreds of miRNAs have been found in eye tissue, which may become a new biomarker for the early diagnosis of non-invasive ocular diseases (22).

#### 3.1.2 Regulation of exosomes secretion

Exosomes are primarily derived from multivesicles formed by the invagination of lysosomal granules in cells, and the outer membranes of multivesicular vesicles are released into the extracellular environment after fusion with the cell surface. Although many cells are able to secrete exosomes under normal or abnormal conditions, under pathological conditions, exosome secretions may increase or their content may change.

On the one hand, it has been found that the RAB family (a member of the RAS oncogene family) of small GTPase proteins controls different steps of vesicle transport in cells (23), such as vesicle budding, mobility of vesicles and organelle interaction through the cytoskeleton, and the junction of vesicles with target chambers to form membrane fusion (24). Since the first proteomic study, endosomal-related members of this family have been identified in exosomes (25). For example, RAB-11 has been implicated in the control of TfR and Hsc70 released from exosomes in K562 cells (26).

On the other hand, in some studies, cellular stress enhanced exosome release (27–29). For example, studies found that radiotherapy-induced cellular senescence is associated with a significant rise in the release of exosome-like microvesicles. In premature aging, this new secretory phenotype depends on p53 activation. Radiation therapy can induce increased DNA damage, such as p53-dependent vesicle increase (28). At present, the specific mechanism of increased secretion caused by cellular stress is unclear, but the increased secretion may act on adjacent cells, leading to pathological changes in these cells.

### 3.2 Regulation of endothelial function

The two main cellular components of retinal microvessels are pericytes and ECs. The formation, maturation, and stabilization of microvessels require the interaction of these two cell types. Endothelial dysfunction is among the risk variables for DR development. Studies have shown that pericytes activated by the hypoxia-inducible factor (HIF) pathway can secrete exosomes under hypoxia and can regulate EC migration, germination, and angiogenesis (30). In addition to those from pericytes, exosomes from neurons, glial cells, ECs, and the circulation can modulate EC integrity and intercellular cross-talk in the neurovascular unit under physiological and pathological conditions (31–33). Gong et al. found that exosomes from mesenchymal stem cells (MSCs) were found to facilitate the transfer of miRNA from MSCs to human umbilical vein ECs (HUVECs) and promote angiogenesis. Their findings demonstrated that MSCs secrete exosomes containing proteins, cytokines, and chemicals that promote HUVEC-mediated tubular formation, increase the bud number of HUVEC spheroids, and attract ECs and promote their proliferation (34). Zhu et al. found that retinal astrocytes may release exosomes to transmit autophagy-inducing signals and regulate EC proliferation and migration; thus, they participate in the occurrence and development of retinal vascular-related diseases (35).

Endothelial-mesenchymal transition (EndMT) has been found to contribute to pathological fibrosis in proliferative DR (PDR). Gu et al. discovered that miR-202-5p secreted by retinal pigment epithelial cells (ARPE) can act as an important mediator of intercellular cross-talk and transfer miR-202-5p *via* the TGF/Smad pathway to inhibit EndMT (36). Cao et al. reported that MSC-derived exosomal SNHG7 can inhibit EndMT and tube formation by human retinal microvascular ECs (HRMECs) stimulated by high glucose (HG) by interacting with the miR-34a-5p/XBP1 signaling pathway, providing a viable treatment approach for DR therapy (37).

### 3.3 Smooth muscle cell proliferation and differentiation

Vascular smooth muscle cells (VSMCs) are a special cell type with abnormal plasticity in response to environmental stressors. Because abnormally increased VSMC proliferation is associated with a variety of vascular disorders, controlling its phenotype may have important implications for delaying DR development. In DR development caused by microvascular diseases caused by microcirculation disorders, hypoxia frequently occurs. Hypoxia promotes VSMC growth. New research suggests that miRNAs are key regulators of the VSMC hypoxia response. Previous studies have shown that miR-1260b is among the more upregulated hypoxia-related amines in VSMCs (38). GDF11-Smad–dependent signaling mediated by miR-1260b is an important signaling method for VSMC proliferation, and hypoxia controls this axis, which promotes aberrant VSMC proliferation (39). The new findings demonstrated that miR-1260b downregulation reduces VSMC proliferation. Consequently, hypoxia-induced increased miR-1260b expression may stimulate VSMC proliferation (39). Research has also shown that HG can induce VSMC calcification/aging, which in turn leads to diabetes-related vascular calcification/aging. Studies have shown that Notch3 is abundant in HG-stimulated HUVECs (HG-HUVEC-Exo). In addition, Notch3 expression in VSMCs was clearly increased in HG-stimulated HUVECs compared with the HG-stimulated HUVEC treatment group. When Notch3 inhibitors act *in vivo* to inhibit Notch3 expression, the capacity of HG-stimulated HUVECs to stimulate calcification/senescence of VSMCs is reduced (40).

Therefore, miR-1260b downregulation can inhibit VSMC proliferation and Notch3 expression, which may consequently delay VSMC calcification/aging and aid in the treatment of DR.

### 3.4 Macrophage activation

Inflammation is the central component of the pathogenesis of diabetes and metabolic syndrome, particularly in the development of complications. DR is considered a vascular and neurodegenerative disease that develops after periods of inadequate blood glucose control. Retinal microvascular disease is the early pathogenesis, which is caused by low-level, persistent leukocyte activation, resulting in recurrent capillary blockage and gradual retinal-depleting ischemia. At the molecular level, macrophage-restricted protein tyrosine phosphatase 1B (PTP1B) is a critical moderator of metabolic syndrome inflammation involving insulin resistance. PTP1B imbalance may underlie retinal microangiopathy (41). Hyperglycemia in patients with diabetes leads to an increased generation of reactive oxygen species (ROS) and the accumulation of advanced glycation or lipid oxidation end products (AGE and ALE, respectively), affecting the physiological functions of the retina (42). AGE and AGE receptor (RAGE) interaction induces a pro-inflammatory phenotype in microglia, resulting in an enhanced release of inflammatory cytokines (TNF-α and IL-6) (43, 44). Elevated TNF-α and IL-1β levels are consistent with increased intraretinal neovascularization in DR and increased microvascular degeneration in ischemic retinopathy (45, 46). Hyperglycemia could activate VEGF expression, and HIF-1 is translocated to the extracellular signal–regulated kinase (ERK)1/2–nuclear factor κB (NF-κB) signaling pathway in the nucleus and microglia (47, 48). VEGF overexpression contributes to retinal neovascularization, whereas translocation of HIF-1 increases the transcription of angiogenesis-related genes (49).

### 3.5 Platelet activation

Vascular fibrosis is the main pathological feature of the proliferative stage of DR. However, the molecular mechanism of its occurrence remains unclear. Connective tissue growth factor (CTGF), a main fibrotic factor, is highly expressed in DR and plays a key role in retinal endothelial membrane thickening (50). The study showed that exosomes of platelet plasma in diabetic rats (DM-PRP-Exos) considerably elevated Müller cell growth and metastasis compared with exosomes of platelet plasma from normal control rats (Nor-PRP-Exos). The above results suggest that platelet plasma secretion–induced fibrogenesis may be triggered by activating the phosphoinositide-3 kinase-serine/threonine kinase (PI3K/Akt) signaling pathway (51). Therefore, under hyperglycemia, platelets are stimulated to produce PRP-Exos, which activate the PI3K/Akt signaling pathway (52).

Platelet plasma exosomes (PRP-Exos) mediate hyperglycemia-induced retinal endothelial damage *via* upregulating the TLR4 signaling pathway. They can transfer platelet cytokines, alter protein expression, and cause retinal endothelial dysfunction and early DR. A study found that the PRP-Exo levels in the circulation of diabetic rats were significantly increased. At the same time, it was further proved that HG effectively enhances the capacity of platelets to produce PRP-Exos *in vitro*. It was shown that PRP-Exos can promote TLR4 expression and that of its downstream proteins MyD88, p-NF-κB/P65, and NF-κB/P65 and activate the TLR4 pathway (53). Accumulating evidence suggests that TLR4 has a vital function in regulating retinal homeostasis and is engaged in DR progression (54). The new study found that CXCL10 may activate the TLR4 pathway *in vitro*. CXCL10 blockade can downregulate the TLR4 signaling pathway and diminish PRP-Exo–induced retinal inflammation. These results suggested that CXCL10 appears to be a key regulator of PRP-Exo–derived retinal endothelial damage in DR (53). Consequently, inhibiting the TLR4 signaling pathway provides a new therapeutic idea for reducing the early vascular DR complications induced by PRP-Exo.

### 3.6 In summary

**Figure 1:**
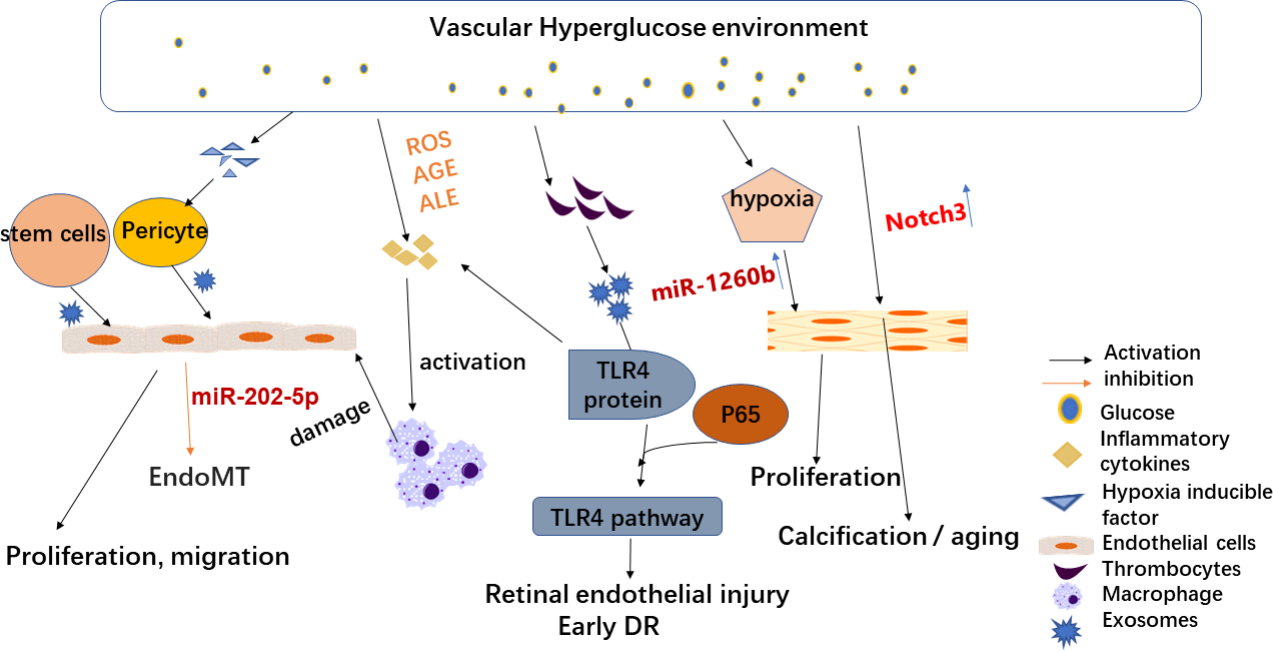
Effects of exosomes on diabetic retinopathy-related cells. High-glucose environment and hypoxia alter circulating exosomes and play a key role in DR development. Therefore, stem cell exosomes may be an effective treatment for DR. Hypoxia-induced increased secretion of hypoxia-inducible factors can lead to endothelial dysfunction (ED) and promote endothelial cell permeability *in vitro*. Hypoxia also leads to changes in circulating exosomes (such as miR-1260b), resulting in the proliferation of vascular smooth muscle cells. A high-glucose microenvironment also leads to a greater generation of reactive oxygen species (ROS) and the build-up of advanced glycation or lipid oxidation end products (AGE or ALE), which in turn leads to inflammatory increased cytokines (TNF-α, the secretion of IL-6) and thus to inflammation and exacerbates the harm of DR. PRP-Exos participate in early DR formation *via* mediating hyperglycemia-induced retinal endothelial damage by upregulating the TLR4 signaling pathway.

As described above, circulating exosome action on various related cells leads to DR. **Figure 1** shows the effect of exosomes on cells associated with DR.

## 4 Effects of exosomes on pericytes

Long-term hyperglycemia causes DR, a frequent microvascular condition. Intercommunication of pericytes and ECs is critical for maintaining vascular homeostasis and remodeling. Hypoxia was found to upregulate circEhmt1 production in pericytes, which can subsequently be transported to ECs by exosomes. In addition, circEhmt1 overexpression has a protective effect against HG-induced EC damage *in vitro*. Mechanistically, circEhmt1, which is strongly expressed in pericyte nuclei, upregulates NFIA (transcription factor) levels and inhibits NLRP3-mediated inflammatory bodies (55).

Diabetes-related stress enhanced cPWWP2A expression in pericytes but had no effect on ECs, according to the findings. Pericytes create cPWWP2A, which is delivered to ECs through exosomes. *In vitro* studies demonstrated that cPWWP2A directly regulates pericyte biology, whereas EC biology is directly regulated by cPWWP2A-carrying exosomes.

CPWWP2A acts as an endogenous miR-579 sponge, isolating and inhibiting the activity of miR-579. Diabetes-induced retinal vascular dysfunction was reduced *in vivo* when cPWWP2A overexpression or miR-579 suppression was used. Furthermore, suppressing cPWWP2A or overexpressing miR-579 can exacerbate microvascular dysfunction by inhibiting the cPWWP2A-mediated signaling pathway. This study suggests that intervention at the level of cPWWP2A or miR-579 expression could provide an opportunity for the treatment of diabetic microvascular complications (2).

Exosomes reduce pericyte migration by downregulating NF-κB p65 signaling, thereby maintaining the function of the blood spinal cord barrier (BSCB). Studies have found that bone marrow MSC–derived extracellular vesicles prevent pericyte migration by inhibiting the activation of the NF-κB signaling pathway. This improves the integrity of the BSCB (56).

Pericyte activation is a key pathogenic characteristic of interstitial fibrosis (RIF). It was found that MSC-derived exosomes deliver the miR-34c-5p by inhibiting core focusing (CF) to reduce cellular activation and help exosomal miR-34c-5p enter pericytes through the RIFcd81–epidermal growth factor receptor (EGFR) ligand receptor complex. The findings of this study offer a potential therapy option for renal fibrosis (57).

## 5 Possible mechanisms of exosome-mediated diabetic retinopathy

Although studies have demonstrated exosome involvement in DR progression, including multiple cascades and interconnections, the specific processes remain unknown. The mechanisms associated with exosomes and DR are mainly hypoxia, inflammation, and stress intensification. As mentioned above, exosomes are extensively involved in DR development and progression. Next, we will attempt to explain the signaling pathways and molecular mechanisms by which exosomes, mainly through miRNAs, may be involved in DR development and progression.

### 5.1 TGF-β–mediated pathways

TGF-β and TGF-β–mediated signaling pathways play an exacerbating role in DR pathogenesis (58). Further research has revealed that they are essential regulators of cell growth and mid-differentiation. For example, as key inducers of tissue fibrosis, they can promote fibroblast proliferation, ultimately leading to tissue fibrosis (59, 60). Lou et al. reported that the miR-21 expression level in the retina of rats with DR was considerably higher than the normal rats, implying that abnormal retinal miR-21 expression may participate in DR pathogenesis. Moreover, the analysis results of this study showed that TGF-β signaling pathway inhibitors greatly reduced the effect of miR-21 in DR in rats and improved ocular hemodynamics in these animals. This suggests that miR-21 controls the TGF-β signaling pathway involved in the pathogenesis of DR. Therefore, TGF-β signaling pathway regulation by miR-21 affects hemodynamics in rats with PDR (61). Li et al. found that inhibiting the TGF-β2/Smad pathway increased miR-200a-3p, which prevented DR development. It was also found that miR-200a-3p was significantly downregulated in both ARPE-19 cells and retinal tissues of rats with DR after HG treatment, whereas TGF-β2 expression was upregulated. Subsequently, miR-200a-3p overexpression greatly accelerated cell proliferation, decreased apoptosis, and reduced the level of secreted inflammatory cytokines and VEGF in HG-injured ARPE-19 cells. MiR-200a-3p overexpression attenuated HG-induced damage in ARPE-19 cells, decreased the secretion of inflammatory cytokines, and downregulated VEGF expression through inactivation of the TGF-β2/Smad pathway. *In vivo*, miR-200a-3p upregulation improved retinal angiogenesis and inflammation in DR in rats, thus providing a novel target for DR therapy. However, it is unclear how miR-200a-3p expression was upregulated *in vivo (* 62).

### 5.2 PI3K/Akt signaling pathway

The PI3K/Akt signaling pathway is a downstream signal of a variety of cell-surface receptors that regulate cell proliferation, survival, and death (63). Zhang et al. discovered that miR-183 was markedly upregulated in a rat model of DR with activation of the PI3K/Akt/VEGF signaling pathway. It was observed that miR-183 expression was upregulated and BTG1 expression was downregulated in retinal tissue in DR in rats. MiR-183 overexpression activated the PI3K/Akt/VEGF signaling pathway to inhibit BTG1 and promote EC proliferation but inhibit apoptosis. According to the findings, miR-183 inhibition could inhibit vascular EC proliferation and angiogenesis by downregulating BTG1 and inactivating the PI3K/Akt/VEGF signaling pathway (64).

It is known that, in PDR, miR-21 expression is increased and can promote retinal pigment epithelial cell proliferation and migration (65). Lu et al. found that miR-21 may be a target for DR therapy because it has the potential to block DR. Downregulation of miR-21 disrupts the survival of retinal vascular ECs (RVECs), inducing apoptosis of these cells, and attenuates angiogenesis by inhibiting the PI3K/Akt/VEGF signaling pathway and upregulating phosphatase and tensin homolog (PTEN). The results indicated that miR-21 overexpression may activate the PI3K/Akt/VEGF signaling pathway by inhibiting PTEN expression, thereby stimulating RVEC activity and angiogenesis in DR in rats, suggesting that miR-21 may be a target of DR treatment (66).

According to Wang et al., HG decreased the relative miR-199a-3p expression level in HRMECs and apre-19 cells but increased VEGF expression. Upregulation of miR-199a-3p not only significantly alleviated HG-induced cell proliferation and migration but also significantly inhibited the PI3K/Akt signaling pathway and HG-induced angiogenesis. MiR-199a-3p upregulation can control the PI3K/Akt pathway by suppressing VEGF and promoting HG-induced angiogenesis in HRMECs (67).

### 5.3 p38 MAPK signaling pathway

The MAPK signaling pathway is present in many cells, delivering extracellular stimuli that elicit biological responses. The P38 signal transduction pathway (MAPK pathway) regulates a wide range of biological functions (68). Li et al. found that miR-141-3p inhibited retinal angiogenesis in glaucoma mice by preventing activation of the docking protein 5 (DOK5)–mediated MAPK signaling pathway. The DOK5 gene was repressed by miR-141-3p, which activated the MAPK pathway. The findings revealed that miR-141-3p reduced the proliferation and angiogenesis of retinal vascular epithelial cells and promoted RGC apoptosis (69). Chen et al. found that MSC-derived exosomes prevented hypoxia-induced cell death by carrying miR-21 and inhibiting p38 MAPK signaling (70). MSC exosomes may aid patients with diabetes, according to their findings. Dai et al. reported that baicalin (BAI) inhibited the activation of the NF-κB and p38 MAPK pathways by upregulating miR-145 and had a protective effect on HG-induced injury of human retinal pigment epithelial cells (71).

### 5.4 NF-κB pathway

NF-κB functions primarily in biological processes, including the inflammatory response and immunity. It was demonstrated to be activated in the diabetic retina in numerous studies, its activation increasing capillary apoptosis (72), which is a precursor to the development of DR characteristics. The activity of the NF-κB signaling pathway has been reported to be enhanced in diabetic rat studies. In turn, NF-κB p65 expression upregulation increases ROS production, which leads to microaneurysms, retinal neovascularization, and vitreous hemorrhage in diabetic rats, and promotes DR progression (73, 74).

According to Li et al., the number of pericytes in retinal capillaries of miR-874 mimics-treated DR in rats rose, whereas EC proliferation was reduced. MiR-874 inhibitor exacerbates DR in diabetic rats after treatment. The results showed that miR-874 overexpression suppressed NF-κB signaling pathway expression and alleviated DR in diabetic rats (75). Li et al. demonstrated low miR-486-3p expression and high TLR4 and NF-κB expression in HG-treated Müller cells. TLR4 is the action site of miR-486-3p. In HG-treated Müller cells, miR-486-3p upregulation or TLR4 downregulation prevented oxidative damage, inflammation, and apoptosis while promoting proliferation. This study highlighted that the protective role of exosomal-induced miR-486-3p upregulation in DR mice is by TLR4/NF-κB axis inhibition (76).

Hui et al. found that miR-145 was downregulated in HG-treated retinal microvascular ECs (RECs), while miR-145 overexpression suppressed enhanced TLR4 expression and NF-κB p65 nuclear translocation in HG-treated RECs. More importantly, miR-145 overexpression reduced REC apoptosis, oxidative stress, and inflammatory cytokine release in HG environments. These findings revealed that miR-145 may exert antioxidant and anti-inflammatory effects in DR (54). Ye and Steinle reported decreased miR-146a expression in human RECs cultured with HG. Overexpression of miR-146a with a miR-146a mimic reduced TLR4/NF-κB and TNF expression in RECs induced by HG. Overexpression of miR-146a in RECs reduced MyD88-dependent and -independent signaling under HG conditions. The results showed that miR-146a suppressed TLR4/NF-κB and TNF-α, the potential site of action for reducing REC inflammation (77).

### 5.5 Summary

**Figure 2:**
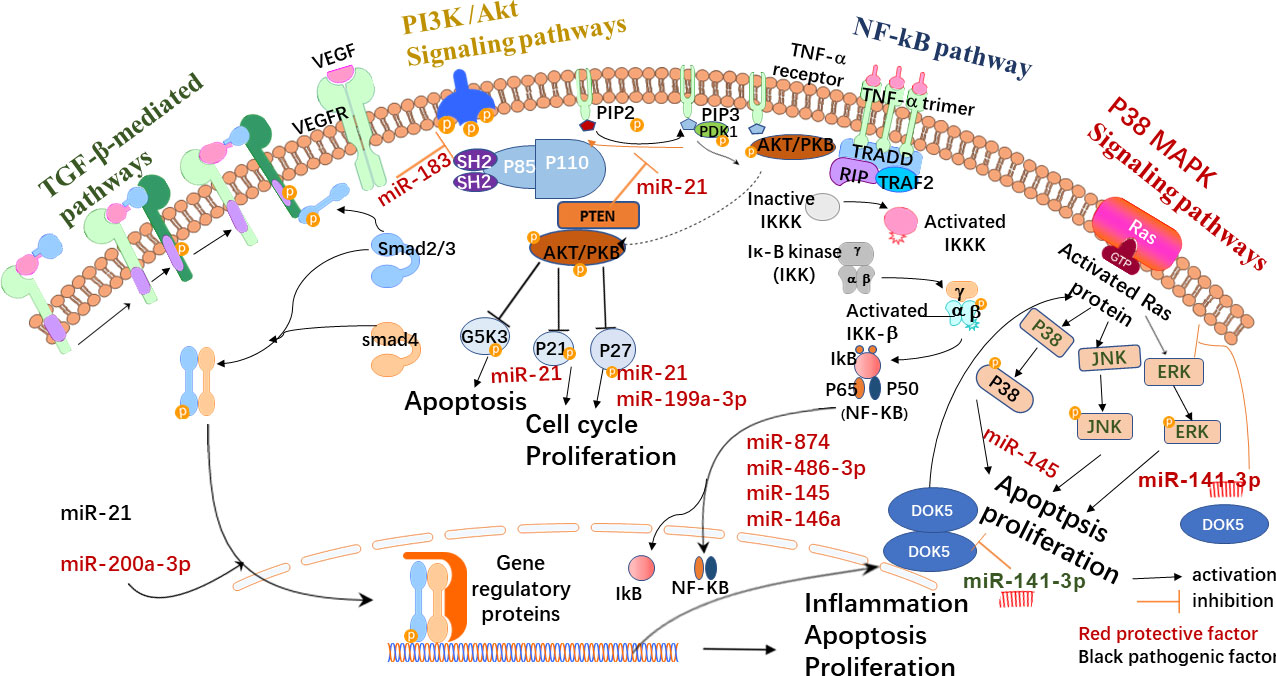
Possible exosome-mediated signaling pathways in DR. Exosomes are synthesized in various cells. Under physiological or pathological conditions, their carriers may change and participate in the formation of the collective pathological state. They may activate TGF-β, the signaling pathway that mediates the hemodynamics of individuals with PDR. By blocking TGF-β, the activation of the signaling pathway promotes cell proliferation and reduces apoptosis and the inflammatory response. Exosomes (such as miR-141-3p) may also inhibit retinal neovascularization by preventing the activation of the p38/MAPK signaling pathway. Exosomes can also be detected by the PI3K/Akt signaling pathway, which can induce vascular endothelial cell proliferation while preventing cell apoptosis, as well as cell proliferation and migration of the retinal pigment epithelium cell. The stimulation of the signaling system may also aid in the reduction of HG-induced cell proliferation, migration, and angiogenesis. In diabetic rats, the activity of the NF-κB signaling pathway rose dramatically, and inhibiting the NF-κB signaling pathway can reduce inflammation, apoptosis, and oxidative stress.

Exosomes have multiple mechanisms of action in DR, but the specific mechanisms remain unclear. However, exosomes might have a critical role in this process and its treatment. **Figure 2** shows the possible signaling pathways and molecular mechanisms involving miRNA in DR formation and development. **Table 1** shows exosome-derived proteins and RNA involved in DR.

**Table 1:** List of exocrine derived proteins and RNA involved in diabetic retinopathy.

Classification	Component	Effect	Reference
Protein	SNHG7	EndMT ↓	(37)
	Notch3	Calcification/aging of VSMCs ↓	(40)
RNA	miR–202–5p	EndMT ↓	(36)
	miR–1260b	Abnormal VSMC proliferation ↑	(39)
	miR–21	Proliferation and migration of RPEC↑	(65)
	miR–200a–3p	Cell proliferation↑ Apoptosis↓, Inflammatory cytokines ↓ VEGF ↓	(62)
	miR–183	Endothelial cell proliferation ↑ Apoptosis↓	(64)
	miR–199a–3p	Neovascularization ↓	(67)
	miR–141–3p	Retinal neovascularization ↓	(69)
	miR–145	RECs Apoptosis↓ oxidative stress↓ Inflammatory cytokines↓	(54, 71)
	miR–874	Number of retinal capillary pericytes↑ Endothelial cell proliferation↓	(75)
	miR–486–3p	Oxidative stress↓ inflammation ↓ Apoptosis↓	(75)
	miR–146a	REC inflammation↓	(77)

## 6 Exosomes and diabetes-related cardiovascular and cerebrovascular events

**Figure 3:**
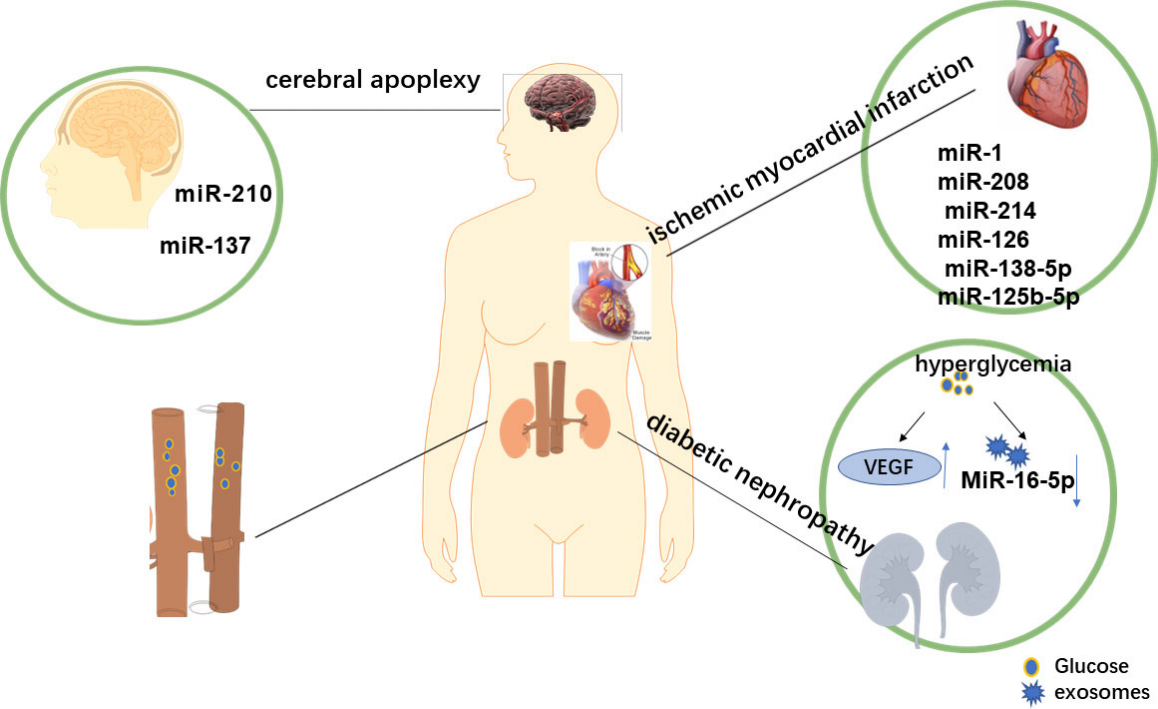
Exocrine-related complications of diabetes. During the development t of diabetes mellitus, changes in the microenvironment also lead to alterations in exocrine bodies, which are involved in cardiovascular and cerebrovascular risk events, such as diabetic nephropathy, myocardial infarction, and stroke. Ever-increasing evidence suggests that the level of exosomes in the blood of patients with diabetes is elevated and is involved in the pathophysiology of diabetes-related diseases. Therefore, we concluded that the overexpression and downregulation of specific miRNA might be a new method to treat diabetes and its related complications.

The vascular complications of diabetes include macrovascular and microvascular complications. In recent years, many studies have proved that miRNA has a good therapeutic effect in the treatment not only of DR but also of other complications of diabetes. There is increasing evidence that exosomes change in the blood of patients with diabetes and are implicated in the progression of diabetes, including microvascular complications, inflammation, and changes in coagulation (78, 79). Next, we briefly introduce the role of miRNA in the treatment of other complications of diabetes. **Figure 3** shows the exosomes related to cardiovascular and cerebrovascular risk events, such as DN, myocardial infarction (MI), and stroke.

### 6.1 Diabetic nephropathy

It was found that rapamycin (mTOR) is a core component of cell growth signaling, and, when its activity is enhanced, it can promote protein translation and autophagy. Autophagy protects against renal injury induced by hyperglycemia (80). Ebrahim et al. found that BMSC-derived exosomes enhanced autophagy *via* blocking the mTOR signaling pathway in a model of DR. In addition, they found that when using MSC-derived exosomes to treat mice with DN, the histological morphology of the kidneys was restored and fibrosis markers were reduced (81).

The development of DN is also associated with podocyte injury (82). VEGF produced by podocytes is not beneficial in treating DN (83). MiR-16-5p could reduce VEGF expression. Hyperglycemia reduces podocyte miR-16-5p production and stimulates VEGF release. Following miR-16-5p overexpression in human embryonic stem cells, exosomes might deliver it to HG-treated podocytes, reducing the degree of podocyte apoptosis and expressing VEGF, thereby delaying the occurrence and development of DN (84).

### 6.2 Ischemic myocardial infarction

MI is a major cause of mortality in all cardiovascular diseases. Ischemia and reperfusion damage is an inevitable adverse reaction after MI. There is presently no effective treatment to reduce the damage to the heart from MI. Recent research has shown that miRNAs in the heart and circulation are markedly altered after MI (85). For example, Cheng et al. found significantly increased miR-1 and miR-208 levels in the urine of patients with acute MI and in the circulating blood of rats after acute MI (86). Mao et al. found that KLF3-AS1 mediates Sirt1 expression by serving as a ceRNA to sponge miR-138-5p, thereby regulating cardiomyocyte pyroptosis and MI progression (87). Furthermore, miR-125b-5p inhibited p53 and BAK1 production, which reduced apoptosis. In addition, increased miR-125b-5p expression in macrophages alleviated hypoxia/reperfusion-induced cellular damage. Enhanced miR-125b-5p production in the myocardium significantly reduced the size of the MI (88, 89). The findings of Zhu et al. showed a unique method, whereby cell-free hypo-exo promotes ischemic heart repair *via* anti-apoptotic miR-125b-5p (89). The role of exosomes in MI is increasingly recognized, but the mechanisms involved and their role in improving cardiac function remain unclear (90).

### 6.3 Stroke

Reflex mechanisms are engaged to protect cerebral perfusion in early hemorrhagic strokes, such as intracerebral hemorrhage and subarachnoid hemorrhage, but the corresponding secondary injury and malfunction can lead to cerebral ischemia, hypoxia, and ultimately neuronal cell death (91). Recent evidence suggests that exosomes may perform various functions in brain repair and as biomarkers for stroke (92). In a previous research, exosome extraction from MSCs was reported to ameliorate specific brain tissue damage in an experimental animal model (93). Exosomes generated by endothelial progenitor cells (EPCs) have been shown to protect ECs from hypoxia/reoxidation injury, which is partially due to the role of miR-210 (94). According to Liu et al., miR-137 upregulation promoted EPC proliferation and angiogenesis in mice with ischemic stroke by the Notch pathway (95). Taken together, exosomes are implicated in the occurrence and progression of stroke through various mechanisms, providing new ideas for stroke treatment.

## 7 Conclusion and prospects

In recent years, exosomes have been studied as a new biological entity engaged in intercellular communication in a variety of physiological and pathological processes. Ongoing technological and experimental advances have the potential to uncover cellular and molecular mechanisms of intercellular communication, organ homeostasis, and disease, enhancing our ability to use these mechanisms as therapeutic and diagnostic tools. DR increases the risk for blindness in those with diabetes. Previous studies have found that exosomes may play a role in both the pathogenesis and treatment of DR. Earlier studies have found that circulatory miRNAs are differentially expressed in subjects with diabetes (96), suggesting that miRNAs could be used as new biomarkers for detecting or predicting the overall progression of the disease, as well as the progression of retinopathy from mild to sight-threatening (97–99). For example, miR-221, an antiangiogenic miRNA found in blood as a biomarker for DR in individuals with T2DM and PDR, was found to be implicated in the physiopathology of T2DM and macrovascular problems (100–102). RNA sequencing has established the potential biomarkers let-7a and miR-151 in serum for early-stage and late-stage DR in patients with T2DM. The previous study has discovered that using multiple miRNAs and anti-miRNAs in a combinational therapy is a unique method that involves targeting numerous pathways with different drugs and may provide a synergistic angiostatic effect that can help to prevent DR pathologic angiogenesis problems. Overexpression of miR-216a, for example, protected against HRMEC damage in DR by inhibiting the NOS2/JAK/STAT axis in the DR rat retina (103). Simultaneously, miRNA-29b-3p increases HRMEC apoptosis in DR by inhibiting SIRT1 (104). Upregulation of miR-203a-3p, which targets VEGFA and HIF-1, may decrease retinal neovascularization in the oxygen-induced retinopathy rat model (105). In DR development, the involvement of exosomes and the role of pericytes have separately been widely studied. However, limited studies have investigated the role of pericyte-related exosomes in the occurrence and development of DR, particularly its biological mechanism. Consequently, future studies should focus on the effect of exocytosis on pericytes and thus its potential role in DR. The use of stem cell exosomes for the treatment of DR requires further basic and clinical research.

In conclusion, exosomes, particularly their miRNAs, are involved in the pathophysiological process of DR and establish multilevel connections. Exosomes have broad application prospects in the treatment and prognostic evaluation of DR.

## Author contributions

S-rN is responsible for writing the manuscript. J-mH is responsible for data collection. YH and SL are mainly responsible for reviewing and revising the article. All authors contributed to the article and approved the submitted version.

## Funding

This work was supported by the Science and Technology Bureau of Quanzhou (grant number 2020CT003 and 2021N036S).

## Conflict of interest

The authors declare that the research was conducted in the absence of any commercial or financial relationships that could be construed as a potential conflict of interest.

## Publisher’s note

All claims expressed in this article are solely those of the authors and do not necessarily represent those of their affiliated organizations, or those of the publisher, the editors and the reviewers. Any product that may be evaluated in this article, or claim that may be made by its manufacturer, is not guaranteed or endorsed by the publisher.

## Abbreviations

DR, diabetic retinopathy; miRNA, microRNA; ECs, endothelial cells; MVBs, multivesicular bodies; miRNAs, microRNAs; NC RNA, noncoding RNA; MSCs, mesenchymal stem cells; HRMECs, human retinal microvascular endothelial cells; HG, high glucose; HUVECs, human umbilical vein endothelial cells; EndMT, endothelial mesenchymal transformation; VSMCs, vascular smooth muscle cells; HUVEC-Exo, human umbilical vein endothelial cell exosome; ROS, reactive oxygen species; AGE, advanced glycation end products; ALE, advanced lipid oxidation end products; RAGE, AGE receptor; CTGF, connective tissue growth factor; PRP-Exos, exosomes derived from platelet-rich plasma; CXCL10, CXCchemokineligand-10; BSCB, Blood spinal cord barrier; BMSC-EVs, Extracellular vesicles derived from bone marrow mesenchymal stem cells; TGF-β 2, transforming growth factor-β 2; VEGF, vascular endothelial growth factor; REC, retinal microvascular endothelial cells; DKD, development of diabetic nephropathy; IR, ischemia and reperfusion; MI, myocardial infarction; EPCs, endothelial progenitor cells.

## References

[1] Cui X Zhu L Zhai R Zhang B Zhang F. Mesenchymal stem cell-derived exosomes: a promising vector in treatment for diabetes and its microvascular complications. Am J Transl Med (2021) 13(5):3942–53. PMC8205700 34149991

[2] Liu C Ge HM Liu BH Dong R Shan K Chen X. Targeting pericyte-endothelial cell crosstalk by circular RNA-cPWWP2A inhibition aggravates diabetes-induced microvascular dysfunction. Proc Natl Acad Sci U.S.A. (2019) 116(15):7455–64. doi: 10.1073/pnas.1814874116 PMC6462073 30914462

[3] van Dijk CG Nieuweboer FE Pei JY Xu YJ Burgisser P van Mulligen E. The complex mural cell: pericyte function in health and disease. Int J Cardiol (2015) 190:75–89. doi: 10.1016/j.ijcard.2015.03.258 25918055

[4] Arboleda-Velasquez JF Valdez CN Marko CK D’Amore PA. From pathobiology to the targeting of pericytes for the treatment of diabetic retinopathy. Curr Diabetes Rep (2015) 15(2):573. doi: 10.1007/s11892-014-0573-2 PMC5599150 25620405

[5] Huang H. Pericyte-endothelial interactions in the retinal microvasculature. Int J Mol Sci (2020) 21(19):7413. doi: 10.3390/ijms21197413 PMC7582747 33049983

[6] Akbari Kordkheyli V Amir Mishan M Khonakdar Tarsi A Mahrooz A Rezaei Kanavi M Hafezi-Moghadam A. MicroRNAs may provide new strategies in the treatment and diagnosis of diabetic retinopathy: Importance of VEGF. Iran J Basic Med Sci (2021) 24(3):267–79. doi: 10.22038/ijbms.2021.52164.11807 PMC8087853 33995938

[7] Hammes HP Feng Y Pfister F Brownlee M. Diabetic retinopathy: targeting vasoregression. Diabetes (2011) 60(1):9–16. doi: 10.2337/db10-0454 21193734 PMC3012202

[8] Winkler EA Bell RD Zlokovic BV. Central nervous system pericytes in health and disease. Nat Neurosci (2011) 14(11):1398–405. doi: 10.1038/nn.2946 PMC4020628 22030551

[9] Frank R. Diabetic retinopathy. New Engl J Med (2004) 350(1):48–58. doi: 10.1056/NEJMra021678 14702427

[10] Hammes H Lin J Wagner P Feng Y Vom Hagen F Krzizok T. Angiopoietin-2 causes pericyte dropout in the normal retina: evidence for involvement in diabetic retinopathy. J Clin Med (2004) 53(4):1104–10. doi: 10.2337/diabetes.53.4.1104 15047628

[11] Li AF Sato T Haimovici R Okamoto T Roy S. High glucose alters connexin 43 expression and gap junction intercellular communication activity in retinal pericytes. Invest Ophthalmol Vis Sci (2003) 44(12):5376–82. doi: 10.1167/iovs.03-0360 14638741

[12] Monickaraj F McGuire P Das A. Cathepsin d plays a role in endothelial-pericyte interactions during alteration of the blood-retinal barrier in diabetic retinopathy. FASEB J (2018) 32(5):2539–48. doi: 10.1096/fj.201700781RR PMC7001867 29263022

[13] McGuire PG Rangasamy S Maestas J Das A. Pericyte-derived sphingosine 1-phosphate induces the expression of adhesion proteins and modulates the retinal endothelial cell barrier. Arterioscler Thromb Vasc Biol (2011) 31(12):e107–115. doi: 10.1161/ATVBAHA.111.235408 PMC3225006 21940944

[14] Caporali A Martello A Miscianinov V Maselli D Vono R Spinetti G. Contribution of pericyte paracrine regulation of the endothelium to angiogenesis. Pharmacol Ther (2017) 171:56–64. doi: 10.1016/j.pharmthera.2016.10.001 27742570

[15] He C Zheng S Luo Y Wang B. Exosome theranostics: Biology and translational medicine. Theranostics (2018) 8(1):237–55. doi: 10.7150/thno.21945 PMC5743472 29290805

[16] Zhang J Li S Li L Li M Guo C Yao J. Exosome and exosomal microRNA: trafficking, sorting, and function. Genomics Proteomics Bioinf (2015) 13(1):17–24. doi: 10.1016/j.gpb.2015.02.001 PMC4411500 25724326

[17] Bosch S Young NA Mignot G Bach JM. Epigenetic mechanisms in immune disease: The significance of toll-like receptor-binding extracellular vesicle-encapsulated microRNA. Front Genet (2020) 11:578335. doi: 10.3389/fgene.2020.578335 33193698 PMC7662563

[18] Fabbri M Paone A Calore F Galli R Gaudio E Santhanam R. MicroRNAs bind to toll-like receptors to induce prometastatic inflammatory response. Proc Natl Acad Sci U.S.A. (2012) 109(31):E2110–2116. doi: 10.1073/pnas.1209414109 PMC3412003 22753494

[19] Ambros V. The functions of animal microRNAs. Nature (2004) 431(7006):350–5. doi: 10.1038/nature02871 15372042

[20] Doench JG Sharp PA. Specificity of microRNA target selection in translational repression. Genes Dev (2004) 18(5):504–11. doi: 10.1101/gad.1184404 PMC374233 15014042

[21] Sevignani C Calin GA Siracusa LD Croce CM. Mammalian microRNAs: a small world for fine-tuning gene expression. Mamm Genome (2006) 17(3):189–202. doi: 10.1007/s00335-005-0066-3 16518686 PMC2679635

[22] Guo R Shen W Su C Jiang S Wang J. Relationship between the pathogenesis of glaucoma and miRNA. Ophthalmic Res (2017) 57(3):194–9. doi: 10.1159/000450957 28073110

[23] Kowal J Tkach M Thery C. Biogenesis and secretion of exosomes. Curr Opin Cell Biol (2014) 29:116–25. doi: 10.1016/j.ceb.2014.05.004 24959705

[24] Stenmark H. Rab GTPases as coordinators of vesicle traffic. Nat Rev Mol Cell Biol (2009) 10(8):513–25. doi: 10.1038/nrm2728 19603039

[25] Thery C Boussac M Veron P Ricciardi-Castagnoli P Raposo G Garin J. Proteomic analysis of dendritic cell-derived exosomes: a secreted subcellular compartment distinct from apoptotic vesicles. J Immunol (2001) 166(12):7309–18. doi: 10.4049/jimmunol.166.12.7309 11390481

[26] Savina A Fader CM Damiani MT Colombo MI. Rab11 promotes docking and fusion of multivesicular bodies in a calcium-dependent manner. Traffic (2005) 6(2):131–43. doi: 10.1111/j.1600-0854.2004.00257.x 15634213

[27] Kanemoto S Nitani R Murakami T Kaneko M Asada R Matsuhisa K. Multivesicular body formation enhancement and exosome release during endoplasmic reticulum stress. Biochem Biophys Res Commun (2016) 480(2):166–72. doi: 10.1016/j.bbrc.2016.10.019 27725157

[28] Lehmann BD Paine MS Brooks AM McCubrey JA Renegar RH Wang R. Senescence-associated exosome release from human prostate cancer cells. Cancer Res (2008) 68(19):7864–71. doi: 10.1158/0008-5472.CAN-07-6538 PMC3845029 18829542

[29] Beer L Zimmermann M Mitterbauer A Ellinger A Gruber F Narzt MS. Analysis of the secretome of apoptotic peripheral blood mononuclear cells: Impact of released proteins and exosomes for tissue regeneration. Sci Rep (2015) 5:16662. doi: 10.1038/srep16662 26567861 PMC4645175

[30] Mayo JN Bearden SE. Driving the hypoxia-inducible pathway in human pericytes promotes vascular density in an exosome-dependent manner. Microcirculation (2015) 22(8):711–23. doi: 10.1111/micc.12227 PMC4715585 26243428

[31] Zagrean AM Hermann DM Opris I Zagrean L Popa-Wagner A. Multicellular crosstalk between exosomes and the neurovascular unit after cerebral ischemia. therapeutic implications. Front Neurosci (2018) 12:811. doi: 10.3389/fnins.2018.00811 30459547 PMC6232510

[32] Huang C Fisher KP Hammer SS Navitskaya S Blanchard GJ Busik JV. Plasma exosomes contribute to microvascular damage in diabetic retinopathy by activating the classical complement pathway. Diabetes (2018) 67(8):1639–49. doi: 10.2337/db17-1587 PMC6054433 29866771

[33] Xu B Zhang Y Du XF Li J Zi HX Bu JW. Neurons secrete miR-132-containing exosomes to regulate brain vascular integrity. Cell Res (2017) 27(7):882–97. doi: 10.1038/cr.2017.62 PMC5518987 28429770

[34] Gong M Yu B Wang J Wang Y Liu M Paul C. Mesenchymal stem cells release exosomes that transfer miRNAs to endothelial cells and promote angiogenesis. Oncotarget (2017) 8(28):45200–12. doi: 10.18632/oncotarget.16778 PMC5542178 28423355

[35] Zhu L Zang J Liu B Yu G Hao L Liu L. Oxidative stress-induced RAC autophagy can improve the HUVEC functions by releasing exosomes. J Cell Physiol (2020) 235(10):7392–409. doi: 10.1002/jcp.29641 PMC7496456 32096219

[36] Gu S Liu Y Zou J Wang W Wei T Wang X. Retinal pigment epithelial cells secrete miR-202-5p-containing exosomes to protect against proliferative diabetic retinopathy. Exp Eye Res (2020) 201:108271. doi: 10.1016/j.exer.2020.108271 33007305

[37] Cao X Xue LD Di Y Li T Tian YJ Song Y. MSC-derived exosomal lncRNA SNHG7 suppresses endothelial-mesenchymal transition and tube formation in diabetic retinopathy *via* miR-34a-5p/XBP1 axis. Life Sci (2021) 272:119232. doi: 10.1016/j.lfs.2021.119232 33600866

[38] Lee J Heo J Kang H. miR-92b-3p-TSC1 axis is critical for mTOR signaling-mediated vascular smooth muscle cell proliferation induced by hypoxia. Cell Death Differ (2019) 26(9):1782–95. doi: 10.1038/s41418-018-0243-z PMC6748132 30518907

[39] Seong M Kang H. Hypoxia-induced miR-1260b regulates vascular smooth muscle cell proliferation by targeting GDF11. BMB Rep (2020) 53(4):206–11. doi: 10.5483/BMBRep.2020.53.4.136 PMC7196185 31818357

[40] Lin X Li S Wang YJ Wang Y Zhong JY He JY. Exosomal Notch3 from high glucose-stimulated endothelial cells regulates vascular smooth muscle cells calcification/aging. Life Sci (2019) 232:116582. doi: 10.1016/j.lfs.2019.116582 31220525

[41] Forrester JV Kuffova L Delibegovic M. The role of inflammation in diabetic retinopathy. Front Immunol (2020) 11:583687. doi: 10.3389/fimmu.2020.583687 33240272 PMC7677305

[42] Sharma Y Saxena S Mishra A Saxena A Natu SM. Advanced glycation end products and diabetic retinopathy. J Ocul Biol Dis Infor (2012) 5(3-4):63–9. doi: 10.1007/s12177-013-9104-7 PMC3709028 24596941

[43] Chen J Sun Z Jin M Tu Y Wang S Yang X. Inhibition of AGEs/RAGE/Rho/ROCK pathway suppresses non-specific neuroinflammation by regulating BV2 microglial M1/M2 polarization through the NF-kappaB pathway. J Neuroimmunol (2017) 305:108–14. doi: 10.1016/j.jneuroim.2017.02.010 28284330

[44] Subedi L Lee JH Gaire BP Kim SY. Sulforaphane inhibits MGO-AGE-Mediated neuroinflammation by suppressing NF-kappaB, MAPK, and AGE-RAGE signaling pathways in microglial cells. Antioxidants (Basel) (2020) 9(9):792. doi: 10.3390/antiox9090792 PMC7554773 32859007

[45] Rivera JC Sitaras N Noueihed B Hamel D Madaan A Zhou T. Microglia and interleukin-1beta in ischemic retinopathy elicit microvascular degeneration through neuronal semaphorin-3A. Arterioscler Thromb Vasc Biol (2013) 33(8):1881–91. doi: 10.1161/ATVBAHA.113.301331 23766263

[46] Yoshida S Yoshida A Ishibashi T. Induction of IL-8, MCP-1, and bFGF by TNF-alpha in retinal glial cells: implications for retinal neovascularization during post-ischemic inflammation. Graefes Arch Clin Exp Ophthalmol (2004) 242(5):409–13. doi: 10.1007/s00417-004-0874-2 15029502

[47] Zhang T Ouyang H Mei X Lu B Yu Z Chen K. Erianin alleviates diabetic retinopathy by reducing retinal inflammation initiated by microglial cells *via* inhibiting hyperglycemia-mediated ERK1/2-NF-kappaB signaling pathway. FASEB J (2019) 33(11):11776–90. doi: 10.1096/fj.201802614RRR PMC6902687 31365278

[48] Yu Z Zhang T Gong C Sheng Y Lu B Zhou L. Erianin inhibits high glucose-induced retinal angiogenesis *via* blocking ERK1/2-regulated HIF-1alpha-VEGF/VEGFR2 signaling pathway. Sci Rep (2016) 6:34306. doi: 10.1038/srep34306 27678303 PMC5039671

[49] Hachana S Pouliot M Couture R Vaucher E. Diabetes-induced inflammation and vascular alterations in the goto-kakizaki rat retina. Curr Eye Res (2020) 45(8):965–74. doi: 10.1080/02713683.2020.1712730 31902231

[50] Kang GY Bang JY Choi AJ Yoon J Lee WC Choi S. Exosomal proteins in the aqueous humor as novel biomarkers in patients with neovascular age-related macular degeneration. J Proteome Res (2014) 13(2):581–95. doi: 10.1021/pr400751k 24400796

[51] Jin X Zhao W Zhou P Niu T. YAP knockdown inhibits proliferation and induces apoptosis of human prostate cancer DU145 cells. Mol Med Rep (2018) 17(3):3783–8. doi: 10.3892/mmr.2017.8352 29286134

[52] Zhang W Jiang H Kong Y. Exosomes derived from platelet-rich plasma activate YAP and promote the fibrogenic activity of Muller cells *via* the PI3K/Akt pathway. Exp Eye Res (2020) 193:107973. doi: 10.1016/j.exer.2020.107973 32059976

[53] Zhang W Dong X Wang T Kong Y. Exosomes derived from platelet-rich plasma mediate hyperglycemia-induced retinal endothelial injury *via* targeting the TLR4 signaling pathway. Exp Eye Res (2019) 189:107813. doi: 10.1016/j.exer.2019.107813 31560926

[54] Hui Y Yin Y. MicroRNA-145 attenuates high glucose-induced oxidative stress and inflammation in retinal endothelial cells through regulating TLR4/NF-kappaB signaling. Life Sci (2018) 207:212–8. doi: 10.1016/j.lfs.2018.06.005 29883722

[55] Ye L Guo H Wang Y Peng Y Zhang Y Li S. Exosomal circEhmt1 released from hypoxia-pretreated pericytes regulates high glucose-induced microvascular dysfunction *via* the NFIA/NLRP3 pathway. Oxid Med Cell Longev (2021) 2021:8833098. doi: 10.1155/2021/8833098 33815662 PMC7994074

[56] Lu Y Zhou Y Zhang R Wen L Wu K Li Y. Bone mesenchymal stem cell-derived extracellular vesicles promote recovery following spinal cord injury via improvement of the integrity of the blood-spinal cord barrier. Front Neurosci (2019) 13:209. doi: 10.3389/fnins.2019.00209 30914918 PMC6423165

[57] Hu X Shen N Liu A Wang W Zhang L Sui Z. Bone marrow mesenchymal stem cell-derived exosomal miR-34c-5p ameliorates RIF by inhibiting the core fucosylation of multiple proteins. Mol Ther (2022) 30(2):763–81. doi: 10.1016/j.ymthe.2021.10.012 PMC8821970 34678513

[58] Kim DJ Kang JM Park SH Kwon HK Song SJ Moon H. Diabetes aggravates post-ischaemic renal fibrosis through persistent activation of TGF-beta1 and shh signalling. Sci Rep (2017) 7(1):16782. doi: 10.1038/s41598-017-16977-z 29196746 PMC5711892

[59] Kilari S Yang B Sharma A McCall DL Misra SJ. Increased transforming growth factor beta (TGF-β) and pSMAD3 signaling in a murine model for contrast induced kidney injury. Sci Rep (2018) 8(1):1–12. doi: 10.1038/s41598-018-24340-z 29700311 PMC5919895

[60] van der Velden JL Wagner DE Lahue KG Abdalla ST Lam YW Weiss DJ. TGF-beta1-induced deposition of provisional extracellular matrix by tracheal basal cells promotes epithelial-to-mesenchymal transition in a c-jun NH2-terminal kinase-1-dependent manner. Am J Physiol Lung Cell Mol Physiol (2018) 314(6):L984–97. doi: 10.1152/ajplung.00053.2017 PMC6032072 29469614

[61] Lou H Wang S Guo T Yang Y. Role of miR-21 in rats with proliferative diabetic retinopathy *via* TGF-β signaling pathway. Eur Rev Med Pharmacol Sci (2019) 23(3 Suppl):9–16. doi: 10.26355/eurrev_201908_18621 31389569

[62] Xue L Xiong C Li J Ren Y Zhang L Jiao K. miR-200-3p suppresses cell proliferation and reduces apoptosis in diabetic retinopathy via blocking the TGF-β2/Smad pathway. Biosci Rep (2020) 40(11):BSR20201545. doi: 10.1042/BSR20201545 33150936 PMC7689656

[63] Fruman DA Chiu H Hopkins BD Bagrodia S Cantley LC Abraham RT. The PI3K pathway in human disease. Cell (2017) 170(4):605–35. doi: 10.1016/j.cell.2017.07.029 PMC5726441 28802037

[64] Zhang Z-Z Qin X-H Zhang J. Metabolism. MicroRNA-183 inhibition exerts suppressive effects on diabetic retinopathy by inactivating BTG1-mediated PI3K/Akt/VEGF signaling pathway. Am J Physiol Endocrinol Metab (2019) 316(6):E1050–60. doi: 10.1152/ajpendo.00444.2018 30835506

[65] Usui-Ouchi A Ouchi Y Kiyokawa M Sakuma T Ito R Ebihara N. Upregulation of mir-21 levels in the vitreous humor is associated with development of proliferative vitreoretinal disease. PloS One (2016) 11(6):e0158043. doi: 10.1371/journal.pone.0158043 27351379 PMC4924816

[66] Lu JM Zhang ZZ Ma X Fang SF Qin XH. Repression of microRNA-21 inhibits retinal vascular endothelial cell growth and angiogenesis *via* PTEN dependent-PI3K/Akt/VEGF signaling pathway in diabetic retinopathy. Exp Eye Res (2020) 190:107886. doi: 10.1016/j.exer.2019.107886 31759996

[67] Wang L Liu W Huang XJE. MicroRNA-199a-3p inhibits angiogenesis by targeting the VEGF/PI3K/AKT signalling pathway in an *in vitro* model of diabetic retinopathy. Pathol M (2020) 116:104488. doi: 10.1016/j.yexmp.2020.104488 32622012

[68] Qi R Liu H Wang Q Wang J Yang F Long D. Expressions and regulatory effects of P38/ERK/JNK mapks in the adipogenic trans-differentiation of C2C12 myoblasts. Cell Physiol Biochem (2017) 44(6):2467–75. doi: 10.1159/000486169 29268268

[69] Zhang LQ Cui H Yu YB Shi HQ Zhou Y Liu MJ. MicroRNA-141-3p inhibits retinal neovascularization and retinal ganglion cell apoptosis in glaucoma mice through the inactivation of docking protein 5-dependent mitogen-activated protein kinase signaling pathway. J Cell Physiol (2019) 234(6):8873–87. doi: 10.1002/jcp.27549 30515784

[70] Chen J Chen J Cheng Y Fu Y Zhao H Tang M. Mesenchymal stem cell-derived exosomes protect beta cells against hypoxia-induced apoptosis *via* miR-21 by alleviating ER stress and inhibiting p38 MAPK phosphorylation. Stem Cell Res Ther (2020) 11(1):97. doi: 10.1186/s13287-020-01610-0 32127037 PMC7055095

[71] Dai C Jiang S Chu C Xin M Song X Zhao B. Baicalin protects human retinal pigment epithelial cell lines against high glucose-induced cell injury by up-regulation of microRNA-145. Exp Mol Pathol (2019) 106:123–30. doi: 10.1016/j.yexmp.2019.01.002 30625293

[72] Antzelevitch C. Genetic, molecular and cellular mechanisms underlying the J wave syndromes. Circ J (2012) 76(5):1054–65. doi: 10.1253/circj.CJ-12-0284 PMC3521574 22498570

[73] Chen F Zhang H-Q Zhu J Liu K-Y Cheng H Li G-L. Puerarin enhances superoxide dismutase activity and inhibits RAGE and VEGF expression in retinas of STZ–induced early diabetic rats. Asian Pacific J Trop Med (2012) 5(11):891–6. doi: 10.1016/S1995-7645(12)60166-7 23146804

[74] Yin Y Chen F Wang W Wang H Zhang X. Resolvin D1 inhibits inflammatory response in STZ-induced diabetic retinopathy rats: Possible involvement of NLRP3 inflammasome and NF-κB signaling pathway. Mol Vis (2017) 23:242. 28465656 PMC5398882

[75] Li R Yuan H Zhao T Yan Y Liu Z Cai J. miR-874 ameliorates retinopathy in diabetic rats by NF-kappaB signaling pathway. Adv Clin Exp Med (2021) 30(4):421–30. doi: 10.17219/acem/130602 33913264

[76] Li W Jin L Cui Y Nie A Xie N Liang G. Bone marrow mesenchymal stem cells-induced exosomal microRNA-486-3p protects against diabetic retinopathy through TLR4/NF-kappaB axis repression. J Endocrinol Invest (2021) 44(6):1193–207. doi: 10.1007/s40618-020-01405-3 32979189

[77] Ye EA Steinle JJ. miR-146a attenuates inflammatory pathways mediated by TLR4/NF-kappaB and TNFalpha to protect primary human retinal microvascular endothelial cells grown in high glucose. Mediators Inflammation (2016) 2016:3958453. doi: 10.1155/2016/3958453 PMC4779539 26997759

[78] Freeman D Noren Hooten N Eitan E Green J Mode N Bodogai M. Altered extracellular vesicle concentration, cargo, and function in diabetes. Diabetes (2018) 67(11):2377–88. doi: 10.2337/db17-1308 PMC6198336 29720498

[79] O'Neill S Bohl M Gregersen S Hermansen K O'Driscoll L. Blood-based biomarkers for metabolic syndrome. Trends Endocrinol Metab (2016) 27(6):363–74. doi: 10.1016/j.tem.2016.03.012 27150849

[80] De Rechter S Decuypere JP Ivanova E van den Heuvel LP De Smedt H Levtchenko E. Autophagy in renal diseases. Pediatr Nephrol (2016) 31(5):737–52. doi: 10.1007/s00467-015-3134-2 26141928

[81] Ebrahim N Ahmed IA Hussien NI Dessouky AA Farid AS Elshazly AM. Mesenchymal stem cell-derived exosomes ameliorated diabetic nephropathy by autophagy induction through the mTOR signaling pathway. Cells (2018) 7(12):556. doi: 10.3390/cells7120226 PMC6315695 30467302

[82] Zhang X Song Z Guo Y Zhou M. The novel role of TRPC6 in vitamin d ameliorating podocyte injury in STZ-induced diabetic rats. Mol Cell Biochem (2015) 399(1-2):155–65. doi: 10.1007/s11010-014-2242-9 25292315

[83] Bus P Scharpfenecker M van der Wilk P Wolterbeek R Bruijn JA Baelde HJ. The VEGF-a inhibitor sFLT-1 improves renal function by reducing endothelial activation and inflammation in a mouse model of type 1 diabetes. Diabetologia (2017) 60(9):1813–21. doi: 10.1007/s00125-017-4322-3 PMC5552850 28620823

[84] Duan YR Chen BP Chen F Yang SX Zhu CY Ma YL. Exosomal microRNA-16-5p from human urine-derived stem cells ameliorates diabetic nephropathy through protection of podocyte. J Cell Mol Med (2021) 25(23):10798–813. doi: 10.1111/jcmm.14558 PMC8642687 31568645

[85] Sahoo S Losordo DW. Exosomes and cardiac repair after myocardial infarction. Circ Res (2014) 114(2):333–44. doi: 10.1161/CIRCRESAHA.114.300639 24436429

[86] Cheng Y Wang X Yang J Duan X Yao Y Shi X. A translational study of urine miRNAs in acute myocardial infarction. J Mol Cell Cardiol (2012) 53(5):668–76. doi: 10.1016/j.yjmcc.2012.08.010 PMC4492106 22921780

[87] Mao Q Liang XL Zhang CL Pang YH Lu YX. LncRNA KLF3-AS1 in human mesenchymal stem cell-derived exosomes ameliorates pyroptosis of cardiomyocytes and myocardial infarction through miR-138-5p/Sirt1 axis. Stem Cell Res Ther (2019) 10(1):393. doi: 10.1186/s13287-019-1522-4 31847890 PMC6918658

[88] Wang X Ha T Zou J Ren D Liu L Zhang X. MicroRNA-125b protects against myocardial ischaemia/reperfusion injury *via* targeting p53-mediated apoptotic signalling and TRAF6. Cardiovasc Res (2014) 102(3):385–95. doi: 10.1093/cvr/cvu044 PMC4030511 24576954

[89] Zhu LP Tian T Wang JY He JN Chen T Pan M. Hypoxia-elicited mesenchymal stem cell-derived exosomes facilitates cardiac repair through miR-125b-mediated prevention of cell death in myocardial infarction. Theranostics (2018) 8(22):6163–77. doi: 10.7150/thno.28021 PMC6299684 30613290

[90] Davidson SM Padro T Bollini S Vilahur G Duncker DJ Evans PC. Progress in cardiac research: from rebooting cardiac regeneration to a complete cell atlas of the heart. Cardiovasc Res (2021) 117(10):2161–74. doi: 10.1093/cvr/cvab200 PMC8344830 34114614

[91] Xiao M Li Q Feng H Zhang L Chen Y. Neural vascular mechanism for the cerebral blood flow autoregulation after hemorrhagic stroke. Neural Plast (2017) 2017:5819514. doi: 10.1155/2017/5819514 29104807 PMC5634612

[92] Otero-Ortega L Laso-Garcia F Gomez-de Frutos M Fuentes B Diekhorst L Diez-Tejedor E. Role of exosomes as a treatment and potential biomarker for stroke. Transl Stroke Res (2019) 10(3):241–9. doi: 10.1007/s12975-018-0654-7 30105420

[93] Otero-Ortega L Gomez de Frutos MC Laso-Garcia F Rodriguez-Frutos B Medina-Gutierrez E Lopez JA. Exosomes promote restoration after an experimental animal model of intracerebral hemorrhage. J Cereb Blood Flow Metab (2018) 38(5):767–79. doi: 10.1177/0271678X17708917 PMC5987932 28524762

[94] Ma X Wang J Li J Ma C Chen S Lei W. Loading MiR-210 in endothelial progenitor cells derived exosomes boosts their beneficial effects on Hypoxia/Reoxygeneation-injured human endothelial cells *via* protecting mitochondrial function. Cell Physiol Biochem (2018) 46(2):664–75. doi: 10.1159/000488635 29621777

[95] Liu XL Wang G Song W Yang WX Hua J Lyu L. microRNA-137 promotes endothelial progenitor cell proliferation and angiogenesis in cerebral ischemic stroke mice by targeting NR4A2 through the notch pathway. J Cell Physiol (2018) 233(7):5255–66. doi: 10.1002/jcp.26312 29206299

[96] Zampetaki A Kiechl S Drozdov I Willeit P Mayr U Prokopi M. Plasma microRNA profiling reveals loss of endothelial miR-126 and other microRNAs in type 2 diabetes. Circ Res (2010) 107(6):810–7. doi: 10.1161/CIRCRESAHA.110.226357 20651284

[97] Jimenez-Lucena R Camargo A Alcala-Diaz JF Romero-Baldonado C Luque RM van Ommen B. A plasma circulating miRNAs profile predicts type 2 diabetes mellitus and prediabetes: from the CORDIOPREV study. Exp Mol Med (2018) 50(12):1–12. doi: 10.1038/s12276-018-0194-y PMC6312530 30598522

[98] Jimenez-Lucena R Rangel-Zuniga OA Alcala-Diaz JF Lopez-Moreno J Roncero-Ramos I Molina-Abril H. Circulating miRNAs as predictive biomarkers of type 2 diabetes mellitus development in coronary heart disease patients from the CORDIOPREV study. Mol Ther Nucleic Acids (2018) 12:146–57. doi: 10.1016/j.omtn.2018.05.002 PMC6023857 30195754

[99] Barutta F Bellini S Mastrocola R Bruno G Gruden G. MicroRNA and microvascular complications of diabetes. Int J Endocrinol (2018) 2018:6890501. doi: 10.1155/2018/6890501 29707000 PMC5863305

[100] Martinez B Peplow PV. MicroRNAs as biomarkers of diabetic retinopathy and disease progression. Neural Regener Res (2019) 14(11):1858–69. doi: 10.4103/1673-5374.259602 PMC6676865 31290435

[101] Mammadzada P Bayle J Gudmundsson J Kvanta A Andre H. Identification of diagnostic and prognostic microRNAs for recurrent vitreous hemorrhage in patients with proliferative diabetic retinopathy. J Clin Med (2019) 8(12):2217. doi: 10.3390/jcm8122217 PMC6947310 31847440

[102] Chen S Yuan M Liu Y Zhao X Lian P Chen Y. Landscape of microRNA in the aqueous humour of proliferative diabetic retinopathy as assessed by next-generation sequencing. Clin Exp Ophthalmol (2019) 47(7):925–36. doi: 10.1111/ceo.13554 31081578

[103] Liu Y Xiao J Zhao Y Zhao C Yang Q Du X. microRNA-216a protects against human retinal microvascular endothelial cell injury in diabetic retinopathy by suppressing the NOS2/JAK/STAT axis. Exp Mol Pathol (2020) 115:104445. doi: 10.1016/j.yexmp.2020.104445 32335083

[104] Zeng Y Cui Z Liu J Chen J Tang S. MicroRNA-29b-3p promotes human retinal microvascular endothelial cell apoptosis *via* blocking SIRT1 in diabetic retinopathy. Front Physiol (2019) 10:1621. doi: 10.3389/fphys.2019.01621 32063865 PMC7000655

[105] Han N Xu H Yu N Wu Y Yu L. MiR-203a-3p inhibits retinal angiogenesis and alleviates proliferative diabetic retinopathy in oxygen-induced retinopathy (OIR) rat model *via* targeting VEGFA and HIF-1alpha. Clin Exp Pharmacol Physiol (2020) 47(1):85–94. doi: 10.1111/1440-1681.13163 31408201

